# Educational Mobility Through Marriage and Risk of Cognitive Impairment in Late Life

**DOI:** 10.1093/geroni/igab046.2645

**Published:** 2021-12-17

**Authors:** Rong Fu

**Affiliations:** Siena College, Loudonville, New York, United States

## Abstract

Objectives: Marriage represents a long-term intimate relationship involving high levels of interaction and shared resources. Education, as an inter-individual resource, may influence the health status of an individual and his/her spouse. The aim of this study was to assess the impact of educational mobility through marriage on the risk of cognitive impairment in older adults. Methods: Data were derived from the 2014 wave of the Chinese Longitudinal Healthy Longevity Survey. The final sample included 1,396 married men and 671 married women aged 65 years and older. Cognitive impairment was assessed using the Mini-Mental State Exam (MMSE). The gender-specific effect of educational mobility on the risk of cognitive impairment was tested by logistic regression analyses. Results: Older men who experienced downward educational mobility through marriage had a higher risk of cognitive impairment, when compared to their upwardly mobile counterparts. This association was not observed in women. Having more years of schooling protected both men and women from being cognitive impaired in late life. Discussion: These findings provide further evidence that downward socioeconomic mobility through marriage is associated with adverse health outcomes. Yet, the impact of spousal education on health must be understood through the lens of gender. Potential mechanisms that may link spousal education to cognition over the life course were discussed, including health literacy, health behaviors, and household resources.

